# Calcitriol Concentration in the Early Phase of Myocardial Infarction and Its Relation to Left Ventricular Ejection Fraction

**DOI:** 10.3390/metabo14120686

**Published:** 2024-12-06

**Authors:** Szymon Olędzki, Aldona Siennicka, Dominika Maciejewska-Markiewicz, Ewa Stachowska, Natalia Jakubiak, Radosław Kiedrowicz, Karolina Jakubczyk, Karolina Skonieczna-Żydecka, Izabela Gutowska, Jarosław Kaźmierczak

**Affiliations:** 1 Department of Cardiology, Pomeranian Medical University in Szczecin, 70-111 Szczecin, Poland; 2 Department of Medical Analytics, Pomeranian Medical University in Szczecin, 70-111 Szczecin, Poland; aldona.siennicka@pum.edu.pl; 3 Department of Human Nutrition and Metabolomics, Pomeranian Medical University in Szczecin, 71-460 Szczecin, Poland; ewa.stachowska@pum.edu.pl (E.S.); natalia.jakubiak@pum.edu.pl (N.J.);; 4 Department of Biochemical Science, Pomeranian Medical University in Szczecin, 71-460 Szczecin, Poland; 5 Department of Medical Chemistry, Pomeranian Medical University in Szczecin, 71-111 Szczecin, Poland

**Keywords:** myocardial infarction, vitamin D, calcitriol

## Abstract

Vitamin D deficiency is one of the most common metabolic disorders in the European population. A low level of 25-OH vitamin D3 is related to an elevated risk of myocardial infarction (MI). The aim of our study was to examine the relationship between calcidiol and calcitriol serum concentration and left ventricular ejection fraction early after interventional treatment for acute coronary syndrome. A total of 80 patients diagnosed with MI, who underwent primary percutaneous coronary intervention, were included in the study. Blood samples for calcidiol, calcitriol, and vitamin D-binding protein were obtained 24 h after primary PCI and were measured using an enzyme-linked immunosorbent assay. Only 9% of patients had a proper level of 25-OHD3 in the serum (30–80 ng/mL). A total of 16% of patients revealed a suboptimal concentration of 25-OHD3 (20–30 ng/mL), and in 75% of patients, the concentration of 25-OHD3 was lower than 20 ng/mL. Moreover, patients with left ventricle ejection fraction of <40% had significantly lower concentrations of calcidiol and calcitriol. A low calcitriol serum concentration affects post-MI left ventricle ejection fraction early after myocardial infarction onset. It seems that 1.25(OH)D3 may contribute to acute myocardial infarction; however, there are insufficient clinical trials related to this topic, and the available evidence is mainly from in vitro studies. We hope these preliminary reports will provide a better understanding of post-MI.

## 1. Introduction

Vitamin D deficiency is one of the most common metabolic disorders in the European population [1]. It is associated with numerous adverse effects, including detrimental effects on cardiovascular function and metabolism [2]. Calcidiol (25-dihydroxycholecalciferol, 25OHD_3_) is the main circulating form of vitamin D, serving as a precursor to calcitriol (1,25-dihydroxycholecalciferol, 1,25 (OH)D3), the hormonally active form. Calcitriol is produced in the kidneys by the enzyme 1-α-hydroxylase (CYP27B1). Calcitriol regulates calcium and phosphate homeostasis, crucial for bone health and cardiac contractility. Deficiencies or dysregulation in this pathway can impact cardiac function, particularly through altered myocardial calcium handling and fibrosis regulation [2,3]. Both calcidiol and calcitriol have implications for systolic and diastolic heart function. Calcitriol affects myocardial contractility by regulating intracellular calcium. It also influences myocardial remodeling and may mitigate myocardial fibrosis, which is associated with systolic dysfunction in heart failure with reduced ejection fraction (HFrEF) [3,4]. In heart failure with preserved ejection fraction (HFpEF), characterized by diastolic dysfunction, calcitriol may play a role by modulating inflammation and improving endothelial function, thereby reducing myocardial stiffness. The pathophysiology of HFpEF is linked to inflammation and nitric oxide dysregulation. Calcitriol’s anti-inflammatory and vascular effects could be particularly relevant here. For HFrEF, calcitriol’s regulation of calcium and myocardial energy efficiency can support systolic performance, although the evidence specific to these mechanisms in human studies remains limited [3,4]. A significant number of studies have correlated vitamin D deficiency and cardiovascular events, including an increased risk of developing cardiac arrhythmias, atrial fibrillation, and sudden cardiac death [5,6].

Giovannucci et al. showed that a low level of 25-OH vitamin D3 was related to an elevated risk of myocardial infarction (MI) [7]. Yang et al. found that calcitriol improved cardiac function and reversed cardiac remodeling in post-MI mice [8]. Investigators proved that calcitriol reduced MI-inducted inflammation and cardiac cell apoptosis [8]. Another study on a murine model revealed that paricalcitol, the analog of the active form of vitamin D2, delayed heart failure (HF) progression by the attenuation of intracellular Ca2+ mishandling remodeling, antifibrotic, and antihypertrophic effect [9]. Moreover, the authors suggested that paricalcitol may have potential antiarrhythmic effects by preventing the reduction of K+ current density and long QT interval in a model of established heart failure [7].

On the contrary, clinical trials failed to support the putative role of vitamin D supplementation in reducing the heart failure hospitalization rate or cardiovascular events rate [10]. Discrepancies in results may be due to different study groups or experimental conditions. It remains unclear whether or not calcitriol (the active form of vitamin D) has cardioprotective properties in humans and, if so, in which clinical situations [11].

In our research, we aim to examine the relationship between calcidiol and calcitriol serum concentration and left ventricular ejection fraction early after interventional treatment for acute coronary syndrome.

## 2. Materials and Methods

### 2.1. Patients

Overall, 80 patients (22 women and 58 men) diagnosed with MI, who underwent primary PCI (percutaneous coronary intervention), were included in the study. The exclusion criteria are shown in Table 1. 

The research project was approved by the local ethics committee (KB-006/09/2022, 28 February 2022), and written informed consent was obtained from all participants before the study.

### 2.2. MI Definition

The diagnosis of myocardial infarction was made using the 2018 European Society of Cardiology guidelines and requirements [12]. Both STEMI (ST-elevation myocardial infarction) and NSTEMI (non-ST-elevation myocardial infarction) patients were included.

### 2.3. Biochemical Analysis

Biochemical parameters were performed in the Laboratory of Independent Public Clinical Hospital in Szczecin during routine analysis at hospital admission. Blood samples for calcidiol (25-OHD3), calcitriol (1,25-OH2D3), and vitamin D-binding protein (VDBP) were obtained 24 h after primary PCI and MI diagnosis and were stored at −80 °C for later measurement. 25-(OH)D3, 1,2-(OH)2D3, and VDBP were measured using an enzyme-linked immunosorbent assay kit (EIAab Science Inc., Wuhan 430075, China).

### 2.4. Post-Myocardial Infarction Ejection Fraction

A Transthoracic Echocardiogram (TTE) was performed the day before discharge from the hospital, usually on the fourth day of hospitalization, before 25-(OH)D3, 1,2-(OH)2D3, and VDBP measurement. Left ventricular ejection fraction (LVEF) was calculated using the biplane Simpson method. Based on LVEF, patients were qualified as patients with low ejection fraction (low-EF) or preserved ejection fraction (preserved-EF). The LVEF cut-off value was 40%.

### 2.5. Statistical Analysis

The statistical analysis was performed using “R 4.0.4”. The normality of continuous variables distribution using the Shapiro–Wilk test was evaluated, and non-parametric tests were used. Data are presented as medians and interquartile ranges (IQRs). The Mann–Whitney U test was used to analyze the differences between the groups. Values of *p* < 0.05 were considered statistically significant.

## 3. Results

Table 2 shows the demographic and laboratory parameters of patients with MI. This dataset reflects a typical MI cohort, with mixed systolic function, evidence of systemic inflammation, and modifiable risk factors, like LDL–cholesterol and BMI.

### 3.1. Vitamin D Status Among All Patients

Only 9% of patients had a proper level of 25-OHD3 in the serum (30–80 ng/mL). A total of 16% of patients revealed a suboptimal concentration of 25-OHD3 (20–30 ng/mL), and in 75% of patients, the concentration of 25-OHD3 was lower than 20 ng/mL. Vitamin D status is presented in Table 3.

### 3.2. Patient Subgroup Analysis

Fifteen of the patients had LVEF < 40%. The median LVEF in the subgroup with low-EF was 34% (IQR 11%) vs. 51% (IQR 11%) in the subgroup with preserved-EF. There were no differences in basic biochemical parameters, age, or BMI (Body Mass Index) between subgroups (Table 4).

### 3.3. Vitamin D Status According to LVEF

**Figure 1:**
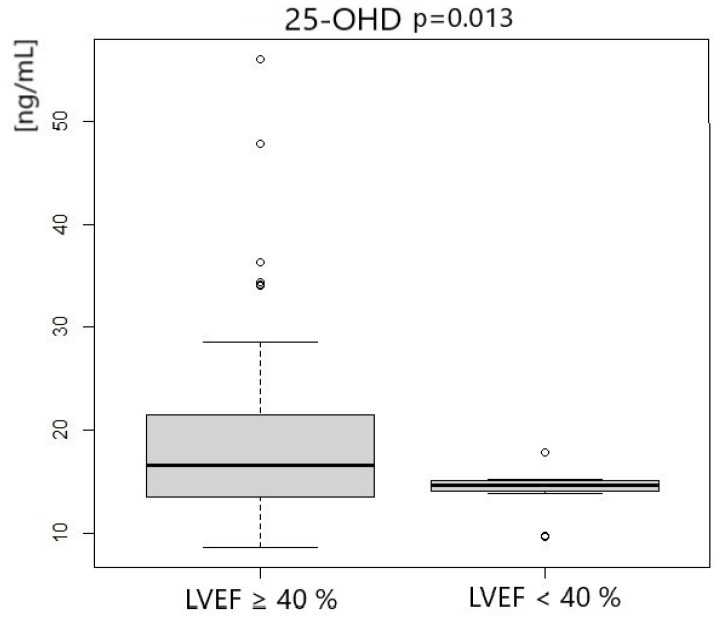
25-OHD concentration according to LVEF measurement.

**Figure 2:**
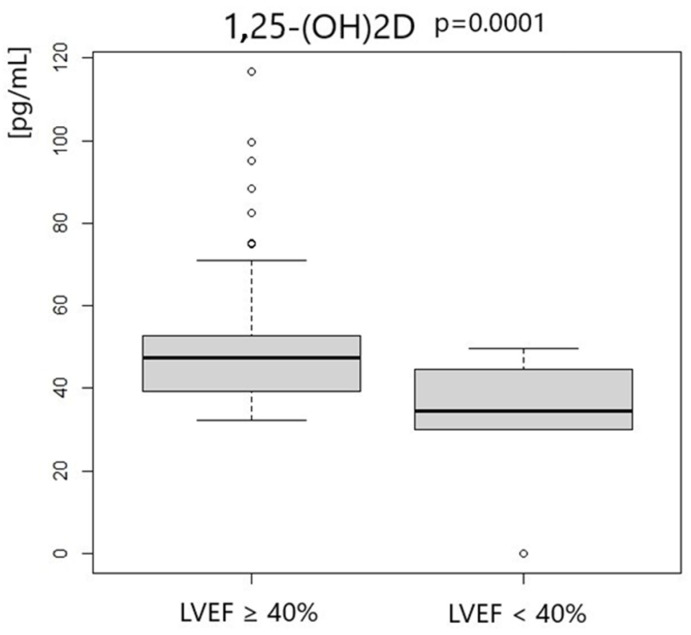
1,25-OH2D concentration according to LVEF measurement.

**Figure 3:**
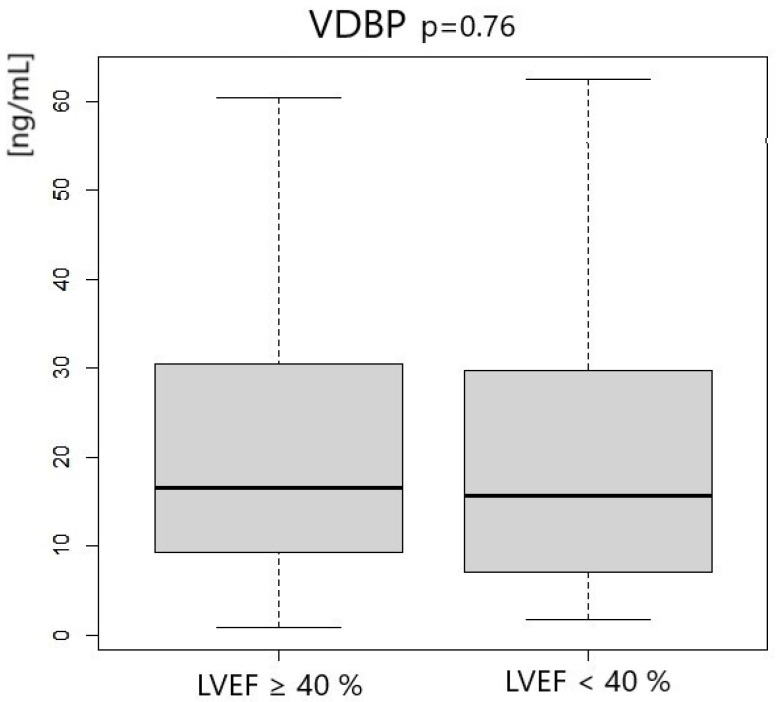
VDBP concentration according to LVEF measurement.

Patients with left ventricle ejection fraction <40% had significantly lower concentrations of 25-OHD3 and 1.25-OH2D3. We did not notice any differences in VDBP concentration. Vitamin D status according to LVEF measurement is presented in Table 5 and Figure 1, Figure 2 and Figure 3.

## 4. Discussion

Many in vitro and pre-clinical studies have provided evidence of the beneficial effects of 1.25(OH)_2_D_3_ on the cardiovascular system [13,14]. Zittermann et al. analyzed 2183 samples from patients scheduled for coronary angiography and 1727 samples from other patients with a wide range of CVDs, including heart transplant candidates, to quantify the association of different parameters with circulating calcitriol. The study revealed that individuals with a lower 1.25(OH)_2_D_3_ concentration have significantly lower concentrations of Fibroblast Growth Factor-23, C-reactive Protein, Intact Parathyroid Hormone, eGFR, and 25-OHD_3_ [15].

The results of our study suggest the potential cardioprotective role of 1.25(OH)_2_D_3_ in acute myocardial infarction. Our research findings are in agreement with previous data obtained from mice experiments. Le et al. found that negative remodeling in 1,25(OH)_2_D_3_-treated mice had been significantly altered 2 weeks after myocardial infarction [16]. The investigators showed that the addition of 1.25(OH)_2_D_3_ to cardiac colony-forming unit fibroblasts (cCFU-Fs) inhibits cell proliferation and attenuates TGF-β-inducted fibroblast differentiation [16]. This may be one of the mechanisms of the cardioprotective effect of calcitriol after MI. Moreover, the calcineurin/NFAT pathway is likely to be a target for the antihypertrophic action of active vitamin D. Tamayo et al. showed that paricalcitol treatment had significantly attenuated transverse aortic constriction-induced left ventricle hypertrophy and left ventricle cavity dilatation, which was related to decreased Rcan1.4 (regulator of calcineurin) gene expression [9]. Other postulated mechanisms of the phenomenon are maintained by the vitamin D extracellular matrix in a dynamic equilibrium by regulating the interaction of matrix metalloproteinases and their inhibitors [17].

In our study, LVEF was assessed early after MI symptoms onset, usually on the fourth day of hospitalization. Thus, the hypothetical effect of low calcitriol serum concentration on the left ventricular contractile was revealed early post-MI. Therefore, the implication of vitamin D active metabolites in neurohumoral activity seems relevant. It was observed that vitamin D has a significant impact on the renin–angiotensin–aldosterone system, which may be crucial in the early phase of post-infarction heart remodeling [18,19,20]. Rapid nongenomic effects of calcitriol in heart cells may also be obtained through direct influence on contractility. Tishkoff et al. found that in adult rat cardiac myocytes, the vitamin D receptor (VDR) is primarily localized to the t-tubule and, thus, may exert an immediate effect on signal transduction mediators and ion channels. Researchers demonstrated that a rapid direct action of 1,25(OH)_2_D_3_ on cardiomyocytes is dependent on the presence of the VDR [21].

Surprisingly, most studies have failed to demonstrate the benefits of vitamin D supplementation in terms of reducing the heart failure hospitalization rate or cardiovascular events rate. We believe that these results cannot be easily transferred to post-MI patients because the research was conducted in a general population of adults [10]. Patients with metabolic chaos during acute MI could potentially benefit from vitamin D supplementation. However, it is known that oral vitamin D supplementation has little effect on 1.25(OH)_2_D_3_ concentrations; therefore, other ways to increase 1.25(OH)_2_D_3_ in acute MI patients should be considered. Further research is needed to explain this phenomenon [22,23,24,25,26,27].

Yang et al. showed that high-dose calcitriol treatment could restore structural impairments and cardiac functions induced by MI in post-MI mice. Researchers have gathered extensive evidence indicating that supplementation with the 1.25(OH)_2_D_3_ may prevent adverse cardiac remodeling caused by MI and may even halt or reverse the development and progression of chronic MI. Mechanistically, the results revealed that calcitriol provides cardioprotective effects by inhibiting cardiac inflammation and preventing the death of cardiomyocytes, which, in turn, reverses the adverse remodeling associated with MI. Additionally, the study demonstrates that 1.25(OH)_2_D_3_ mitigates MI-induced cardiac inflammation through two pathways by suppressing NF-κB signaling via inhibition of p-p65 nuclear translocation and upregulating the expression of the IL-10 gene [8].

However, this study has some limitations. It is important that there were no significant differences in troponin T (TnT) serum concentration between patients with low or preserved EF. TnT levels were measured immediately upon hospital admission, just before PCI. Taking into account dynamic changes in Tnt concentrations during the acute phase of MI, it seems that there were no differences in delay in primary PCI between low-EF and preserved-EF patients. There were also no differences in LDL–cholesterol concentration, BMI, age, glycated hemoglobin range, and GFR. Thus, patients with low and preserved EF did not differ significantly at baseline. Moreover, there are many contributing factors, including those not covered in the exclusion criteria, which can have an impact on MI and vitamin D status. These factors include lifestyle, potassium and sodium balance between heart chambers, genetic predispositions for fat metabolism, heart muscle capacity, diabetes history, hormonal imbalances, vitamin B deficiency, stress, digestive issues, smoking, and inherited autoimmune diseases, such as Familial Mediterranean Fever (FMF) and fibromyalgia. This study does not include important factors, such as amyloid beta concertation, blood pressure, or complete blood count (CBC), which were not measured. These should be included in future investigations.

## 5. Conclusions

This short communication shows that a low serum calcitriol concentration is associated with post-MI severity. It seems that 1.25(OH)_2_D_3_ may contribute to acute myocardial infarction; however, there are insufficient clinical trials related to this topic, and the available evidence is mainly from in vitro studies. We hope that these preliminary reports will provide a better understanding of the post-MI and its implications.

## Figures and Tables

**Table 1:** Exclusion criteria from participation in the study. ALT—Alanine Transaminase, AST—Aspartate Aminotransferase, GFR—Glomerular Filtration Rate.

Previous Health Issues
pregnancy
age under 18 years
chronic inflammation diseases
hematologic diseases
liver diseases—AST or ALT > 150 UI/L
kidney diseases—GFR < 30 mL/min/1.73 m^2^
PCI complications
hypersensitivity reactions to antiplatelet drugs

**Table 2:** Patient characteristics. LVEF—left ventricular ejection fraction; hs-TnT—high-sensitivity troponin T; Hba1c—glycated hemoglobin A1c; LDLs—low-density lipoproteins; WBCs—White Blood Cells; PLTs—platelets; Hgb—hemoglobin; RBCs—Red Blood Cells; GFR—Glomerular Filtration Rate; BMI—Body Mass Index.

Parameters	All Patients	
	Median	IQR
Age [years]	66.5	18.29
LVEF %	49	13.25
Initial hs-TnT [μg/L]	0.11	0.51
Hba1c [%]	5.9	0.62
LDL–cholesterol [mg/dL]	124.5	62.75
WBCs [g/L]	9.98	3.9
PLTs [g/L]	222.5	72.25
Hgb [mmol/L]	9.1	1.1
RBCs [t/L]	4.63	0.69
GFR [mL/min/1.73 m^2^]	82	31
BMI [kg/m^2^]	28.01	5.64

**Table 3:** Vitamin D status among all patients. 25-OHD3—calcidiol; 1,25-OH2D3—calcitriol; VDBP—vitamin D-binding protein.

Vitamin D Status	All Patients	
	Median	IQR
25-OHD3 [ng/mL]	15.49	8.71
1.25-OH2D3 [pg/mL]	45.48	11.75
VDBP [ng/mL]	19.31	21.34

**Table 4:** Patient analysis according to LVEF. LVEF—left ventricular ejection fraction; hs-TnT—high-sensitivity troponin T; Hba1c—glycated hemoglobin A1c; LDLs—low-density lipoproteins; WBCs—White Blood Cells; PLTs—platelets; Hgb—hemoglobin; RBCs—Red Blood Cells; GFR—Glomerular Filtration Rate; BMI—Body Mass Index.

Parameters	LVEF ≥ 40%		LVEF < 40%		*p*
	Median	IQR	Median	IQR	
Age [years]	67	17.5	57	13	0.76
Initial hs-TnT [μg/L]	0.114	0.501	0.213	0.837	0.34
Hba1c [%]	5.8	0.7	5.9	0.325	0.37
LDLs [mg/dL]	132	66	123.5	45	0.38
WBCs [g/L]	9.94	3.79	10.46	5.69	0.25
PLTs [g/L]	221	87	221.5	37.5	0.87
Hgb [mmol/L]	9	1.1	9.3	0.775	0.79
RBCs [t/L]	4.59	0.73	4.82	0.41	0.68
GFR [mL/min/1.73 m^2^]	82	33	81.5	26	0.83
BMI [kg/m^2^]	28.72	5.71	27.23	4.69	0.09

**Table 5:** Vitamin D status according to LVEF. 25-OHD3—calcidiol; 1,25-OH2D3—calcitriol; VDBP—vitamin D-binding protein.

Vitamin D Status	LVEF ≥ 40%		LVEF < 40%		*p*
	Median	IQR	Median	IQR	
**25-OHD3 [ng/mL]**	16.46	7.98	14.61	9.04	0.013
**1,25-OH2D3 [pg/mL]**	47.34	13.35	34.65	12.41	0.0001
VDBP [ng/mL]	16.54	21.18	15.64	22.68	0.76

## Data Availability

Data available on request due to legal an ethical restrictions.

## Abbreviations

1,25 (OH)D3—calcitriol; 25OHD3—calcidiol, ALT—Alanine Transaminase; AST—Aspartate Aminotransferase; BMI—Body Mass Index; CYP27B1—enzyme 1-α-hydroxylase; CBC—complete blood count; CRP—c-reactive protein; F-23—Fibroblast Growth Factor-23; GFR—Glomerular Filtration Rate; Hba1c—glycated hemoglobin A1c; HF—heart failure; HFrEF—heart failure with preserved ejection fraction; HFrEF—heart failure with reduced ejection fraction; Hgb—hemoglobin; hs-TnT—high-sensitivity troponin T; IL-10—Interleukin 10; IQRs—interquartile ranges; PLT—platelets; LDLs—low-density lipoproteins; low-EF—low ejection fraction; LVEF—left ventricular ejection fraction; MI—myocardial infarction; NF-κB—nuclear factor kappa-light-chain-enhancer of activated B cells; NSTEMI—non-ST-elevation myocardial infarction; PCI—percutaneous coronary intervention; preserve-EF—preserve ejection fraction; PTH—parathyroid hormone; RBCs—Red Blood Cells; STEMI—ST-elevation myocardial infarction; TTE—Transthoracic Echocardiogram; VDBP—vitamin D-binding protein; VEF—left ventricular ejection fraction; WBCs—White Blood Cells.

## References

[1] Chanchlani R., Ackerman S., Piva E., Harvey E. (2016). Intraperitoneal Calcitriol for Treatment of Severe Hyperparathyroidism in Children with Chronic Kidney Disease: A Therapy Forgotten. Perit. Dial. Int..

[2] 25-Hydroxyvitamin D Levels and the Risk of Mortality in the General Population–PubMed. https://pubmed.ncbi.nlm.nih.gov/18695076/.

[3] Meems L.M., Brouwers F.P., Joosten M.M., Lambers Heerspink H.J., de Zeeuw D., Bakker S.J., Gansevoort R.T., van Gilst W.H., van der Harst P., de Boer R.A. (2016). Plasma calcidiol, calcitriol, and parathyroid hormone and risk of new onset heart failure in a population-based cohort study. ESC Heart Fail..

[4] Reddy Y.N.V., Lewis G.D., Shah S.J., LeWinter M., Semigran M., Davila-Roman V.G., Anstrom K., Hernandez A., Braunwald E., Redfield M.M. (2017). INDIE-HFpEF (Inorganic Nitrite Delivery to Improve Exercise Capacity in Heart Failure With Preserved Ejection Fraction): Rationale and Design. Circ Heart Fail..

[5] Barsan M., Brata A.M., Ismaiel A., Dumitrascu D.I., Badulescu A.V., Duse T.A., Dascalescu S., Popa S.L., Grad S., Muresan L. (2022). The Pathogenesis of Cardiac Arrhythmias in Vitamin D Deficiency. Biomedicines.

[6] Graczyk S., Grzeczka A., Pasławska U., Kordowitzki P. (2023). The Possible Influence of Vitamin D Levels on the Development of Atrial Fibrillation-An Update. Nutrients.

[7] Giovannucci E., Liu Y., Hollis B.W., Rimm E.B. (2008). 25-Hydroxyvitamin D and Risk of Myocardial Infarction in Men: A Prospective Study. Arch. Intern. Med..

[8] Yang S., Wang C., Ruan C., Chen M., Cao R., Sheng L., Chang N., Xu T., Zhao P., Liu X. (2022). Novel Insights into the Cardioprotective Effects of Calcitriol in Myocardial Infarction. Cells.

[9] Tamayo M., Martín-Nunes L., Val-Blasco A., G.M-Piedras M.J., Navarro-García J.A., Lage E., Prieto P., Ruiz-Hurtado G., Fernández-Velasco M., Delgado C. (2020). Beneficial Effects of Paricalcitol on Cardiac Dysfunction and Remodelling in a Model of Established Heart Failure. Br. J. Pharmacol..

[10] Supplementation With Vitamin D and Omega-3 Fatty Acids and Incidence of Heart Failure Hospitalization: VITAL-Heart Failure.—Abstract—Europe PMC. https://europepmc.org/article/pmc/pmc7054158.

[11] Scragg R., Stewart A.W., Waayer D., Lawes C.M.M., Toop L., Sluyter J., Murphy J., Khaw K.-T., Camargo C.A. (2017). Effect of Monthly High-Dose Vitamin D Supplementation on Cardiovascular Disease in the Vitamin D Assessment Study: A Randomized Clinical Trial. JAMA Cardiol..

[12] Thygesen K., Alpert J.S., Jaffe A.S., Chaitman B.R., Bax J.J., Morrow D.A., White H.D., Executive Group on behalf of the Joint European Society of Cardiology (ESC)/American College of Cardiology (ACC)/American Heart Association (AHA)/World Heart Federation (WHF) Task Force for the Universal Definition of Myocardial Infarction (2018). Fourth Universal Definition of Myocardial Infarction (2018). Glob. Heart.

[13] Ahmadieh H., Arabi A. (2023). Association between Vitamin D and Cardiovascular Health: Myth or Fact? A Narrative Review of the Evidence. Womens Health.

[14] Garcia V.C., Martini L.A. (2010). Vitamin D and Cardiovascular Disease. Nutrients.

[15] Zittermann A., Zelzer S., Herrmann M., Gummert J.F., Kleber M., Trummer C., Theiler-Schwetz V., Keppel M.H., Maerz W., Pilz S. (2024). Determinants of Circulating Calcitriol in Cardiovascular Disease. J. Steroid Biochem. Mol. Biol..

[16] Le T.Y.L., Ogawa M., Kizana E., Gunton J.E., Chong J.J.H. (2018). Vitamin D Improves Cardiac Function After Myocardial Infarction Through Modulation of Resident Cardiac Progenitor Cells. Heart Lung Circ..

[17] Rahman A., Hershey S., Ahmed S., Nibbelink K., Simpson R.U. (2007). Heart Extracellular Matrix Gene Expression Profile in the Vitamin D Receptor Knockout Mice. J. Steroid Biochem. Mol. Biol..

[18] Ferder M., Inserra F., Manucha W., Ferder L. (2013). The World Pandemic of Vitamin D Deficiency Could Possibly Be Explained by Cellular Inflammatory Response Activity Induced by the Renin-Angiotensin System. Am. J. Physiol. Cell Physiol..

[19] Freundlich M., Li Y.C., Quiroz Y., Bravo Y., Seeherunvong W., Faul C., Weisinger J.R., Rodriguez-Iturbe B. (2014). Paricalcitol Downregulates Myocardial Renin-Angiotensin and Fibroblast Growth Factor Expression and Attenuates Cardiac Hypertrophy in Uremic Rats. Am. J. Hypertens..

[20] Luo Q., Yan W., Nie Q., Han W. (2022). Vitamin D and Heart Failure: A Two-Sample Mendelian Randomization Study. Nutr. Metab. Cardiovasc. Dis..

[21] Tishkoff D.X., Nibbelink K.A., Holmberg K.H., Dandu L., Simpson R.U. (2008). Functional Vitamin D Receptor (VDR) in the t-Tubules of Cardiac Myocytes: VDR Knockout Cardiomyocyte Contractility. Endocrinology.

[22] Kimball S.M., Hanwell H.E. (2012). “Calcitriol” Is Not Synonymous with “Vitamin D”. Mult. Scler. Int..

[23] Simpson R.U., Thomas G.A., Arnold A.J. (1985). Identification of 1,25-dihydroxyvitamin D3 receptors and activities in muscle. J. Biol. Chem..

[24] Weishaar R.E., Kim S.N., Saunders D.E., Simpson R.U. (1990). Involvement of vitamin D3 with cardiovascular function. III. Effects on physical and morphological properties. Am. J. Physiol..

[25] Green J.J., Robinson D.A., Wilson G.E., Simpson R.U., Westfall M.V. (2006). Calcitriol modulation of cardiac contractile performance via protein kinase C. J. Mol. Cell. Cardiol..

[26] Weishaar R.E., Simpson R.U. (1987). Vitamin D3 and cardiovascular function in rats. J. Clin. Investig..

[27] Xiang W., Kong J., Chen S. (2005). Cardiac hypertrophy in vitamin D receptor knockout mice: Role of the systemic and cardiac renin-angiotensin systems. Am. J. Physiol. Endocrinol. Metab..

